# Discovery of *Aspergillus frankstonensis* sp. nov. during environmental sampling for animal and human fungal pathogens

**DOI:** 10.1371/journal.pone.0181660

**Published:** 2017-08-09

**Authors:** Jessica J. Talbot, Jos Houbraken, Jens C. Frisvad, Robert A. Samson, Sarah E. Kidd, John Pitt, Sue Lindsay, Julia A. Beatty, Vanessa R. Barrs

**Affiliations:** 1 Sydney School of Veterinary Science, Faculty of Science, The University of Sydney, Camperdown, New South Wales, Australia; 2 Westerdijk Fungal Biodiversity Institute, Utrecht, Netherlands; 3 Department of Biotechnology and Biomedicine, Technical University of Denmark, Kongens Lyngby, Denmark; 4 National Mycology Reference Centre, Microbiology and Infectious Diseases, SA Pathology, Adelaide, South Australia, Australia; 5 CSIRO Food Science, CSIRO, North Ryde, New South Wales, Australia; 6 Faculty of Science and Engineering, Macquarie University, North Ryde, New South Wales, Australia; Leibniz-Institut fur Naturstoff-Forschung und Infektionsbiologie eV Hans-Knoll-Institut, GERMANY

## Abstract

Invasive fungal infections (IFI) due to species in *Aspergillus* section *Fumigati* (ASF), including the *Aspergillus viridinutans* species complex (AVSC), are increasingly reported in humans and cats. The risk of exposure to these medically important fungi in Australia is unknown. Air and soil was sampled from the domiciles of pet cats diagnosed with these IFI and from a nature reserve in Frankston, Victoria, where *Aspergillus viridinutans sensu stricto* was discovered in 1954. Of 104 ASF species isolated, 61% were *A*. *fumigatus sensu stricto*, 9% were AVSC (*A*. *felis*-clade and *A*. *frankstonensis* sp. nov.) and 30% were other species (30%). Seven pathogenic ASF species known to cause disease in humans and animals (*A*. *felis*-clade, *A*. *fischeri*, *A*. *thermomutatus*, *A*. *lentulus*, *A*. *laciniosus A*. *fumisynnematus*, *A*. *hiratsukae*) comprised 25% of isolates overall. AVSC species were only isolated from Frankston soil where they were abundant, suggesting a particular ecological niche. Phylogenetic, morphological and metabolomic analyses of these isolates identified a new species, *A*. *frankstonensis* that is phylogenetically distinct from other AVSC species, heterothallic and produces a unique array of extrolites, including the UV spectrum characterized compounds DOLD, RAIMO and CALBO. Shared morphological and physiological characteristics with other AVSC species include slow sporulation, optimal growth at 37°C, no growth at 50°C, and viriditoxin production. Overall, the risk of environmental exposure to pathogenic species in ASF in Australia appears to be high, but there was no evidence of direct environmental exposure to AVSC species in areas where humans and cats cohabitate.

## Introduction

Aspergillosis is an opportunistic respiratory or systemic disease affecting a range of mammalian, avian and reptile hosts globally. It is most commonly caused by fungi belonging to *Aspergillus* section *Fumigati* (ASF), of which 19 of 63 described species are known to be pathogenic [1, 2]. The saprophytic, ubiquitous fungus *Aspergillus fumigatus sensu stricto* is the most common cause of aspergillosis overall. However, other species within the section, including members of the *Aspergillus viridinutans* species complex (AVSC) are increasingly recognized as emerging causes of invasive fungal infections (IFI). Currently, of the 10 accepted species in the AVSC, six are known pathogens including *A*. *udagawae*, *A*. *felis*, *A*. *wyomingensis*, *A*. *pseudoviridinutans*, *A*. *parafelis and A*. *pseudofelis* [2–8].

*Aspergillus felis*, *A*. *wyomingensis* and *A*. *udagawae* cause an invasive form of fungal rhinosinusitis in systemically immunocompetent cats called sino-orbital aspergillosis [2, 4]. Infection is often fatal, with high MICs of azole antifungals recorded *in vitro* and clinical resistance *in vivo* [4, 9]. *Aspergillus felis* has been isolated from clinical samples in humans, cats, dogs and birds, demonstrating a broad range of susceptible hosts [4]. While feline sino-orbital aspergillosis has been reported in diverse geographic regions including Europe, the United States and Japan, the majority of cases are from Australia [4, 10]. Other, less common causes of feline fungal rhinosinusitis include *A*. *fumigatus s*. *str*., *A*. *thermomutatus* (syn. *N*. *pseudofischeri*), *A*. *fischeri*, *A*. *lentulus* and *A*. *niger* species complex [9–13].

*Aspergillus felis* was first isolated from infected cats in Australia [4]. It has since been isolated from environmental samples from the USA and from other countries with low to no disease incidence, in soil (Czech Republic, India, Zambia) [14], Caribbean Pine (Sri Lanka) [14], indoor air (Germany) [4] and on desert rocks in Chile [15]. The environmental prevalence of *A*. *felis* and other pathogenic species in section *Fumigati* in Australia has not been investigated, so environmental risk factors for exposure are unknown.

Here we report the findings of an investigation to determine if ASF species causing IFI in cats could be isolated from the home environments of cats with these infections. We also collected environmental samples from the region where *A*. *viridinutans s*. *str*. was first recovered and discovered a novel species, *Aspergillus frankstonensis* sp. nov.

## Materials and methods

### Sampling and isolation

With the permission of private land owners, as per the University of Sydney Human Ethics Approval (project number 2014/980), soil and air samples were collected from yards and gardens where eight cats previously diagnosed with SOA due to *A*. *udagawae* (n = 2), *A*. *felis* (n = 5) and *A*. *wyomingensis* (n = 1) were domiciled (5 in New South Wales (NSW), 2 in Victoria (VIC), 1 in the Australian Capital Territory (ACT). These locations included urban (n = 6), rural (n = 1) and semi-rural (n = 1) properties. Samples were also collected from municipal nature reserves in the coastal town of Frankston in the Mornington Peninsula region, VIC, where *A*. *viridinutans s*. *str*. was originally isolated in soil and rabbit dung [16]. Permission to sample was granted by the Frankston City Council. Field studies did not involve endangered or protected species.

A minimum of two air samples were collected at each site directly onto dichloran rose-bengal agar (DRBC) (Thermo Scientific, Thebarton, Australia) using a Merck Millipore MAS-100 NT^®^ air sampler (Merck KGaA, Darmstadt, Germany) at a rate of 100L/minute [17]. Ten soil samples, including garden and lawn soils (sandy and loamy) and mulches (sugar cane, wood chip and straw hay) were collected at each site to a depth of 10 cm and stored at 4°C. Isolation of fungi from soil was achieved by 10^−1^ to 10^−4^ serial dilutions with sterile water. The resulting dilutions were transferred to a Whirlpak^®^ (Nasco) homogenizer bag, heated at 75°C for 30 min, and 0.1 mL was inoculated onto DRBC [14, 18]. Additionally, 17 representative samples of different soil types from each location were processed using the same method without heat treatment.

Samples of commercial cat litter substrates, including the same brands used by cats in the study (recycled paper pellets, clumping clay, recycled timber pellets and silica crystals), were processed as for soil samples and using direct plating. Stored and unopened commercially available dry cat food samples were also tested in the same manner.

Air samples were incubated in the dark for up to three weeks at 37°C. Treated soil samples were cultured on DRBC and malt extract agar supplemented with chloramphenicol (MEASC) [19] for up to three weeks at 37°C. Fungal colonies were sub-cultured onto malt extract agar (MEA) (Thermo Scientific Oxoid Microbiology Products, Thebarton, Australia; Landsmeer, Netherlands) [19] for further analysis.

### Identification of environmental isolates

For environmental isolates with gross macro- and microscopic morphological features consistent with *Aspergillus* spp. (excluding *Aspergillus* section *Nigri* species), amplification and sequencing of the ITS region and partial β-tubulin (*BenA*) gene was performed for species identification as previously described [20, 21]. A BLAST [22] search on GenBank was performed with the newly generated sequences, which were also aligned with *Aspergillus* references sequences (Samson et al. 2014) using *MEGA* version 6 software [23]. Phylogenetic analysis was performed using the maximum likelihood discrete method (tree searching method of 1000 replicate trees) and bootstrapping to determine the statistical support of the nodes.

#### Phylogenetic analysis of AVSC isolates

Nine environmental isolates identified in the AVSC were further examined based on molecular studies and phylogenetic analysis results of less than 100% match for *A*. *felis* (n = 7) and *A*. *viridinutans* (n = 2) ITS and *BenA* sequences. Additional molecular analysis was performed by sequencing partial calmodulin (*CaM*), actin (*Act*) and RNA polymerase II second largest subunit (*RPB2*) genes as previously described [8, 24]. Specific primers targeting the minichromosome maintenance factor gene (*Mcm7*) were developed based on previously published primers [8]: MCM7-709F_Fum ACTCGTGTCTCGGACGTCAAACC (forward) and MCM7-1348R_Fum GATTTGGCRACACCAGGATCACCCAT (reverse). For comparative analysis, these genes were also sequenced for AVSC members in the CBS-KNAW collection and for a new clinical isolate from the USA from a cat with SOA (see Table 1). Phylogenetic and molecular evolutionary analyses were conducted using Randomised Axelerated Maximum Likelihood (RAxML) and Bayesian methods [25, 26].

**Table 1:** Isolates included in phylogenetic analysis of the *Aspergillus viridinutans* complex.

Identification number	Species name	Source	Location	ITS	BenA	Cam	RPB2	Actin	MCM7
**DTO 006-A3**	*A*. *udagawae*	soil	USA	KY808735	KY808572	KY808696	KY808908	KY808509	KY808858
**DTO 019-D7**	*A*. *udagawae*	unknown	unknown	KY808737	KY808573	KY808697	KY808909	KY808510	KY808859
**DTO 019-D8**	*A*. *udagawae*	unknown	unknown	KY808738	KY808574	KY808698	KY808910	KY808511	KY808860
**DTO 019-F2**	*A*. *arcoverdensis*	soil	Australia	KY808747	KY808575	KY808699	KY808911	KY808512	KY808861
**DTO 050-F1, CBS 127.56**^T^**, NRRL 4365**	*A*. *viridinutans*	rabbit dung	Australia	EF669978	AF134779	DQ534162	EF669765	DQ094862	KY808862
**DTO 331-G6, CBS 105.55**^T^**, DTO 052-C8, NRRL 2244**	*A*. *aureolus*	soil	Ghana	EF669950	EF669808	KY808720	KY808943	DQ094861	KY808895
**DTO 131-E3**	*A*. *felis*	cat, RBM	Australia	JX021671	KY808576	KY808701	KY808912	KY808513	KY808863
**DTO 131-E4**	*A*. *felis*	cat, RBM	Australia	JX021673	JX021692	KY808702	KY808913	KY808514	KY808864
**DTO 131-E6, CBS 130244**	*A*. *felis*	cat, RBM	Australia	JX021675	JX021694	JX021717	KY808915	KY808516	KY808866
**DTO 131-E5**	*A*. *felis*	cat, RBM	Australia	JX021674	JX021693	JX021719	KY808914	KY808515	KY808865
**DTO 131-E9**	*A*. *felis*	cat, RBM	Australia	JX021676	JX021696	KY808703	KY808916	KY808517	KY808867
**DTO 131-F1**	*A*. *felis*	cat, RBM	Australia	JX021677	JX021697	KY808704	KY808917	KY808518	KY808868
**DTO 131-F2**	*A*. *felis*	cat, RBM	Australia	JX021678	JX021698	KY808705	KY808918	KY808519	KY808869
**DTO 131-F3**	*A*. *felis*	cat, RBM	Australia	JX021679	JX021699	KY808706	KY808919	KY808520	KY808870
**DTO 131-F4, CBS 130245**^T^	*A*. *felis*	cat, RBM	Australia	J X021685	JX021700	JX021715	KY808920	KY808521	KY808871
**DTO 131-F6**	*A*. *felis*	cat, RBM	Australia	JX021680	JX021702	JX021721	KY808921	KY808522	KY808872
**DTO 131-F9, CBS 130246**	*A*. *felis*	cat, SNC	Australia	JX021681	JX021704	JX021724	KY808922	KY808523	KY808873
**DTO 131-G1**	*A*. *felis*	cat, RBM	Australia	JX021682	JX021705	JX021725	KY808923	KY808524	KY808874
**DTO 131-G2, CBS 130247**	*A*. *felis*	cat, RBM	Australia	JX021683	JX021706	JX021726	KY808924	KY808525	KY808875
**DTO 131-G3, CBS 130248**	*A*. *felis*	cat, RBM	Australia	JX021684	JX021707	JX021727	KY808925	KY808526	KY808876
**DTO 155-G2**	*A*. *wyomingensis*	cat, RBM	Australia	JX021685	JX021709	KY808707	KY808927	KY808527	KY808878
**DTO 155-G3, CBS 130249**	*A*. *felis*	dog, VH	Australia	JX021686	JX021711	JX021713	KY808928	KY808528	KY808879
**DTO 157-D7, CBS 114217**^T^	*A*. *udagawae*	soil	Brazil	AB250781	AF132226	AB748566	KY808929	KY808529	KY808880
**DTO 157-D8, CBS 114218**	*A*. *udagawae*	soil	Brazil	AB250782	AB248303	AY689373	KY808930	KY808530	KY808881
**DTO 159-C9, CBS 130250**	*A*. *felis-*clade	cat, RBM	United Kingdom	JX021689	JX021712	JX021714	KY808931	KY808531	KY808882
**DTO 166-D6**	*A*. *udagawae*	cat	Australia	KY808740	KY808579	KY808710	KY808932	KY808532	KY808883
**DTO 175-H3**	*A*. *sp*.	surface water	Portugal	KY808741	KY808580	KY808711	KY808933	KY808533	KY808884
**DTO 176-F1**	*A*. *felis*	air	Germany	KY808742	KY808581	KC305168	KY808934	KY808534	KY808885
**DTO 278-B6, CBS 137452**^T^	*A*. *siamensis*	soil	Thailand	-	KY808582	AB776704	KY808712	KY829134	KY808886
**DTO 278-B7, CBS 137453**	*A*. *aureolus*	soil	Brazil	KY808743	KY808583	KY808713	KY808935	KY808535	KY808887
**DTO 283-D3**	*A*. *udagawae*	soil	Thailand	KY808744	KY808584	KY808714	KY808936	KY808536	KY808888
**DTO 303-A1**	*A*. *pseudoviridinutans*	*Pinus caribea* (pine tree)	Sri Lanka	KY808745	KY808585	KY808715	KY808937	KY808537	KY808889
**DTO 308-H6**	*A*. *udagawae*	soil	Turkey	KY808746	KY808586	KY808716	KY808938	KY808539	KY808890
**DTO 316-C8**	*A*. *felis/ A*. *conversis*	CBS culture contaminant	The Netherlands	KY808750	KY808587	KY808717	KY808939	KY808540	KY808891
**DTO 316-F7, CBS 139187**^T^	*A*. *arcoverdensis*	semi-desert soil	Brazil	KY808748	AB818845	AB818856	KY808940	KY808541	KY808892
**DTO 316-F9, CBS 139188**	*A*. *arcoverdensis*	unknown	Brazil	KY808749	KY808588	KY808718	KY808941	KY808542	KY808893
**DTO 327-G4**	*A*. *viridinutans*	human patient	The Netherlands	KY808751	KX903288	KY808719	KY808942	KY808543	KY808894
**DTO 332-B1, CBS 135456**^T^	*A*. *wyomingensis*	coal mine reclamation site soil	Glenrock, USA	HG324081	KY808589	KY808721	HF937378	KY808544	KY808896
**NRRL 6106, DTO 342-I3, CBS 140764**	*A*. *pseudoviridinutans*	unknown	Unknown	-	AF134778	KJ914709	KY808965	KY808566	KJ914726
**NRRL 62900, CM-3147, DTO 342-I4, CBS 140762**^T^	*A*. *felis-*clade	human, OPE	Spain	-	KJ914692	KJ914702	KY808966	KY808567	KJ914720
**NRRL 62901, CM-5623, DTO 342-I5, CBS 140765**	*A*. *felis-*clade	human, lungs	Portugal	-	KJ914693	KJ914703	KY808967	KY808568	KJ914721
**NRRL 62902, CM-4518, DTO 342-I6, CBS 140766**	*A*. *felis-*clade	human, nail	Spain	-	KJ914696	KJ914704	KY808968	KY808569	KJ914722
**NRRL 62903, CM-6087, DTO 342-I7, CBS 140763**^T^	*A*. *felis-*clade	human, sputum	Spain	-	KJ914697	KJ914705	KY808969	KY808570	KJ914723
**NRRL 62904, NIHAV1, DTO 304-I5, CBS 140396**^T^	*A*. *pseudoviridinutans*	human, lung	USA	-	GQ144441	GQ144442	KJ914730	KY808538	KJ914727
**DTO 341-E8**	*A*. *felis-*clade	woodland soil	Frankston, Australia	KY808757	KY808595	KY808725	KY808949	KY808550	KY808902
**DTO 341-E9**	*A*. *felis-*clade	woodland soil	Frankston, Australia	KY808758	KY808596	KY808726	KY808950	KY808551	KY808903
**DTO 341-F1**	*A*. *felis-*clade	woodland soil	Frankston, Australia	KY808759	KY808597	KY808727	KY808951	KY808552	KY808904
**DTO 341-E6**	*A*. *felis-*clade	woodland soil	Frankston, Australia	KY808755	KY808593	KY829133	KY808947	KY808548	KY808900
**DTO341-E4**	*A*. *felis-*clade	woodland soil	Frankston, Australia	KY808753	KY808591	KY808723	KY808945	KY808546	KY808898
**DTO 341-F2**	*A*. *felis-*clade	woodland soil	Frankston, Australia	KY808760	KY808598	KY808728	KY808952	KY808553	KY808905
**DTO 341-E5**	*A*. *felis-*clade	woodland soil	Frankston, Australia	KY808754	KY808592	KY829132	KY808946	KY808547	KY808899
**OHIG B6-A1**	*A*. *felis-*clade	cat, RBM	Connecticut, USA	KY808857	KY808695	KY808734	KY808970	KY808571	KY808907
**DTO 341-E7, CBS 142233, IBT 34172**	*A*. *frankstonensis* sp. nov.	woodland soil	Frankston, Australia	KY808756	KY808594	KY808724	KY808948	KY808549	KY808901
**DTO 341-F3, CBS 142234, IBT 34204**^T^	*A*. *frankstonensis* sp. nov.	woodland soil	Frankston, Australia	KY808761	KY808599	KY808729	KY808953	KY808554	KY808906
**DTO 341-E3, CBS 142231**	*A*. *udagawae*	cat, RBM	Kealba, Australia	KY808752	KY808590	KY808722	KY808944	KY808545	KY808897
DTO 153-A1*, CBS 458.75	*A*. sp.	soil	India	KY808736	AY685178	HG426048	KY808926	DQ094853	KY808877

^T^ = Type strain; CBS ID number culture collection of the Westerdijk Fungal Biodiversity Institute, the Netherlands; DTO in-house collection ID number at Westerdijk Institute, the Netherlands; NRRL ID number Agricultural Research Service Culture Collection, USA; OHIG ID number One Health Infectious Disease Research Group Collection, University of Sydney, Australia; RBM = retrobulbar mass; SNC = sino-nasal cavity; VH = vitreous humor; OPE = oropharyngeal exudates.

* = This isolate was included in this study as previous in-house sequence analysis showed close phylogenetic relatedness to members of this complex.— = no sequence or accession number available.

#### Phenotypic species differentiation

The physiology and macro- and micromorphology of the two isolates demonstrated to be phylogenetically distinct from other AVSC species (CBS 142234 and CBS 142233) were studied. Isolates were grown at 25°C on Czapek yeast agar (CYA) [19], Czapek yeast agar with 5% NaCl (CYAS) [27], yeast extract sucrose agar (YES) [19], MEA, oatmeal agar (OAT), creatine sucrose agar (CREA) [19], dichloran 18% glycerol agar (DG18) [28] for seven days. For temperature growth testing isolates were also grown on CYA at 30°C, 37°C, 45°C and 50°C for seven days.

#### Extrolite analysis

Extrolite extraction was performed on the two phylogenetically distinct isolates after growth on CYA and YES agar at 25°C and 37°C for 7 days. Three agar plugs were extracted according to the agar plug extraction method of Smedsgaard [29]. Extracts were analysed using UHPLC-DAD (Dionex Ultramate 3000 UHPLC) and compounds were identified against an internal database of UV spectra and literature [30]. Extrolite standards were available as reported by Nielsen et al. [30].

#### Antifungal susceptibility testing

Antifungal susceptibility testing was performed on all AVSC environmental isolates, and the clinical isolate, using Sensititre YeastOne YO8 microdilution trays (Trek Diagnostic Systems, Thermo Fisher Scientific, Scoresby, Australia) to assess the minimum inhibitory concentration (MIC) values of posaconazole (POS), itraconazole (ITZ), voriconazole (VCZ), fluconazole (FLU), ketoconazole (KCZ), amphotericin B (AMB), and minimum effective concentration (MEC) of caspofungin (CSP) as previously described [31].

#### Mating type analysis

Mating type for all AVSC environmental isolates was determined by targeting the MAT1-1 and MAT1-2 genes [4]. Mating experiments were also performed on isolates with opposite mating types of the same species where available, or with other members of the AVSC where unavailable, on OAT and MEA in the dark at 30°C. Ascospore viability tests were performed by rupturing ascomata, suspending ascospores in 0.05% Tween 80 and heating at 70°C for 60 min. After heating, 100 μL of the ascospore suspension was plated on 2% MEA and incubated at 28°C for 24 h [32]. To act as a negative control, the same treatment was also applied to the conidia of paired parental strains from the mating plate. Scanning electron microscopy was performed on all ascospores (Emitech 550K Sputter coater JEOL 6480LA).

#### Nomenclature

The electronic version of this article in Portable Document Format (PDF) in a work with an ISSN or ISBN will represent a published work according to the International Code of Nomenclature for algae, fungi, and plants, and hence the new names contained in the electronic publication of a PLOS article are effectively published under that Code from the electronic edition alone, so there is no longer any need to provide printed copies.

In addition, new names contained in this work have been submitted to MycoBank from where they will be made available to the Global Names Index. The unique MycoBank number can be resolved and the associated information viewed through any standard web browser by appending the MycoBank number contained in this publication to the prefix http://www.mycobank.org/MB/. The online version of this work is archived and available from the following digital repositories: PubMed Central, LOCKSS.

## Results

### Sequence-based identification

Overall 104 ASF species were isolated from all sites including 61% (n = 64) *A*. *fumigatus s*. *str*., 9% (n = 9) AVSC (*A*. *felis-*clade and *A*. *frankstonensis* sp. nov.) and 30% (n = 31) other ASF species (see S2 Table, S1 Fig) (Genbank accession numbers: KY808753-KY808856 (ITS); KY808591- KY808694 (*BenA*)). Pathogenic ASF species (*A*. *A*. *felis-*clade (n = 7), *A*. *fischeri* (n = 6), *A*. *thermomutatus* (n = 1), *A*. *lentulus* (n = 2), *A*. *laciniosus* (n = 2), *A*. *fumisynnematus* (n = 4), *A*. *hiratsukae* (n = 1) comprised 25% of isolates overall. AVSC species were only isolated from Frankston soil where they were abundant and comprised 41% of isolates from that site.

*Aspergillus* isolates were only recovered from three of the nine sampled locations when no heat treatment was used. Isolated species included *A*. *fumigatus s*. *str*. (Kealba, n = 1), *A*. *hiratsukae* (Frankston, n = 1; Amaroo, n = 1). *Aspergillus fumigatus s*. *str*. was also isolated from recycled paper cat litter. No *Aspergillus* species were isolated from other cat litter types and dry food tested. The clinical isolate from the cat in the US (Table 1) was phylogenetically closely related to the type of *A*. *parafelis*, CBS 140762, in the *A*. *felis-*clade.

#### Phylogeny

**Fig 1:**
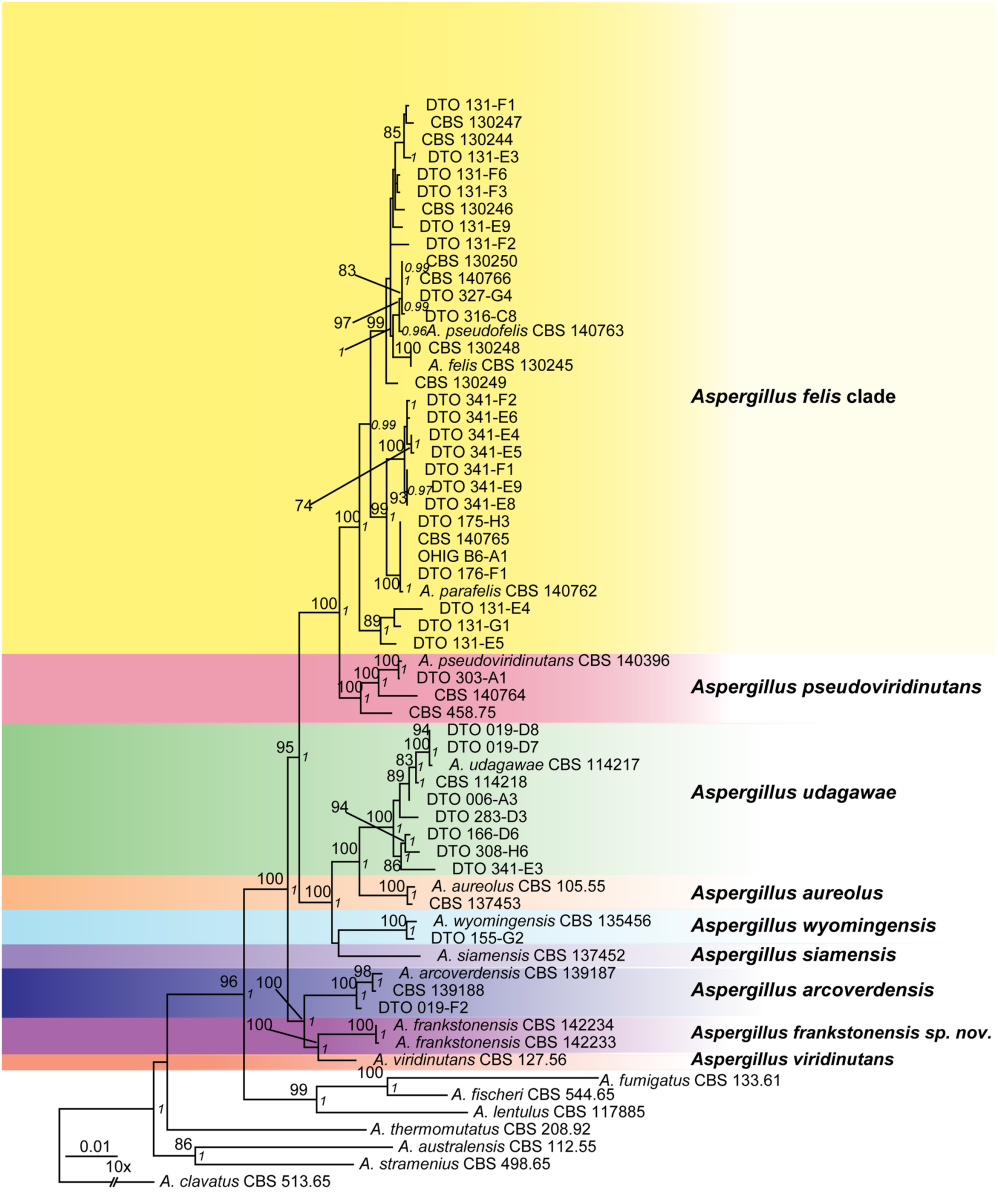
*Aspergillus viridinutans* species complex combined phylogenetic tree. Tree based on sequencing of *Mcm7*, *BenA*, *Act*, *RPB2*, *CaM* genes (phylogeny model Kimura 2, gamma distribution, 1000 bootstrap replicates with Bayesian method posterior probability values in italics). Isolates previously described as *A*. *felis*, *A*. *parafelis* and *A*. *pseudofelis* are listed here under the one grouping “*A*. *felis-*clade*”*.

**Fig 2:**
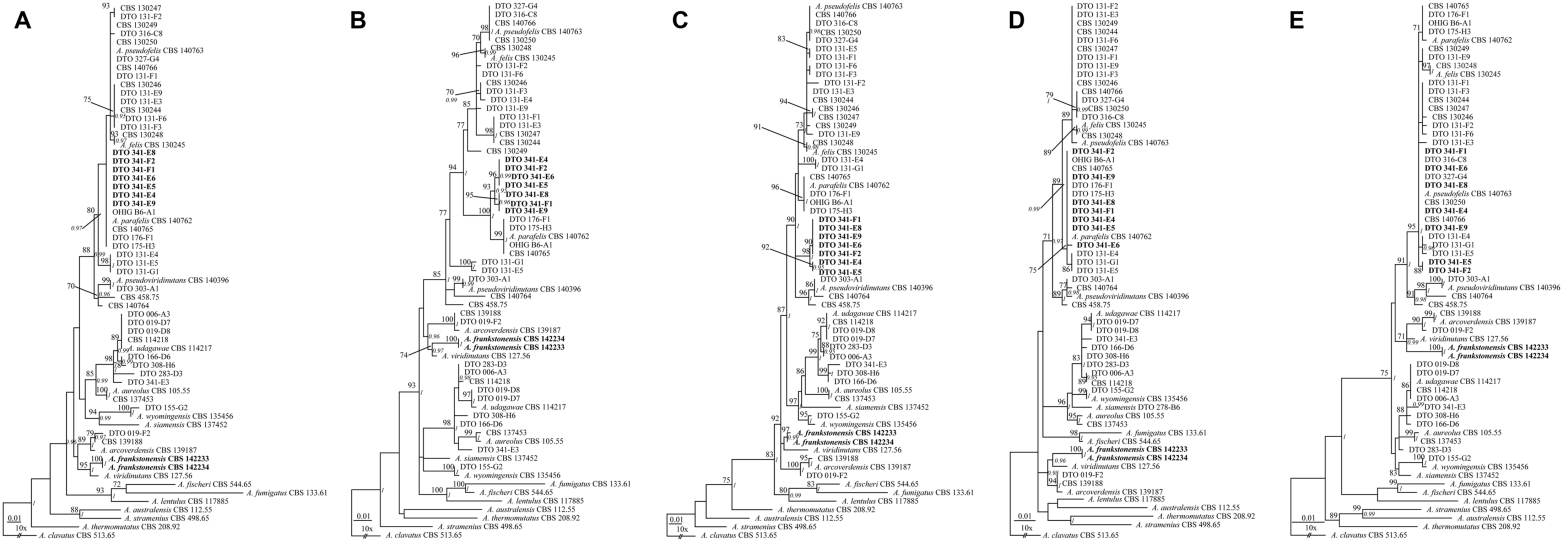
*Aspergillus viridinutans* species complex individual gene phylogenetic trees. All phylogenies made with gamma distribution and 1000 bootstrap replicates with Bayesian method posterior probability values in italics. A = *Act* (phylogeny model General Time Reversible); B = *BenA* (phylogeny model Kimura 2); C = *CaM* (phylogeny model Tamura-Nei); D = *Mcm7* (phylogeny model Kimura 2); E = *RPB2* (phylogeny model General Time Reversible).

The length of the datasets were *BenA* 422 basepairs (bp), *CaM* 485 bp, *Act* 379 bp, *RPB2* 831 bp and *Mcm7* 450 bp. Phylogenetic analysis of combined *BenA*, *CaM*, *Act*, *RPB2* and *Mcm7* data (2567 bp) confirmed seven isolates belong in the *A*. *felis*-clade, related to the type of *A*. *parafelis* CBS 140762, and two isolates were most closely related to, but phylogenetically distinct from *A*. *viridinutans s*. *str*. (Figs 1 and 2). These two isolates had identical sequences and are described as a new species below, *A*. *frankstonensis* sp. nov.

The individual and combined phylogenies (*BenA*, *CaM*, *RPB2*, *Act*, *Mcm7*) show that the 9 isolates from Frankston soil are accommodated in the AVSC and ASF (Figs 1 and 2). Two of these isolates (CBS 142233, CBS 142234) were most closely related to *A*. *viridinutans s*. *str*. in all trees, with bootstrap support (bs) >70% in four of the six trees generated (*RPB2* 71% bs; *Act* 95% bs; *BenA* 74% bs; combined 100% bs). This was also supported by Bayesian method posterior probability values (*RPB2* 0.99 pp; *Act* 1.00 pp; *BenA* 0.97 pp; combined 1.00 pp). *Aspergillus arcoverdensis* takes a basal position to those two species with high statistical support (bs 100%, 1.00 pp). These sequences of CBS 142234 and CBS 142233 are different from the other species in AVSC and ASF, with the genetic change seen in the distance of the horizontal branch. For both these isolates the percentage difference at ITS, *BenA*, *CaM*, *RPB2*, *Act* and *Mcm7* were 99%, 96%, 98%, 99%, 98% and 98% respectively when compared with *A*. *viridinutans s*. *str*. (NRRL 4365). A BLAST analysis did not show a 100% similarity match on GenBank for either of these isolates, and the highest similarities were with the type strain for *A*. *viridinutans s*. *str*. The remaining seven Frankston soil isolates (DTO 341-F2, DTO 341-E6, DTO 341-E4, DTO 341-E5, DTO 341-F1, DTO 341-E9, DTO 341-E8) were also shown to be accommodated in the AVSC and ASF. The results of the analysis of the combined dataset (Fig 1) showed that these isolates were with high statistical support (99% bs, 1.00 pp) most closely related to the type of *A*. *parafelis* CBS 140762. The individual and combined phylogenies also showed that isolate CBS 458.75 is accommodated in the AVSC, most closely related to *A*. *pseudoviridinutans* isolates in five of the six trees generated (*Act* 70% bs, 0.96 pp; *CaM* 96% bs, 1.00 pp; *Mcm7* 89% bs 1.00 pp; *RPB2* 91% bs, 0.98 pp; combined 100% bs, 1.00 pp). Isolates DTO 131-E4, DTO 131-E5 and DTO 131-G1 formed a separate clade to other *A*. *felis* isolates in the *Act* (98% bs, 1.00 pp), *Mcm7* (86% bs, <0.95 pp), *RPB2* (<70% bs, 0.96 pp) and combined trees (89%, 1.00 pp). The *BenA* tree showed DTO 131-G1 and DTO 131-E5 were in the same clade (100% bs, 1.00 pp) and basal to the *A*. *felis*-clade, while DTO 131-E4 was more closely related to the type of *A*. *felis* (CBS 130245) (70% bs, 0.99 pp). In the *CaM* tree, DTO 131-E4 and DTO 131-G1 were in the same clade (100% bs, 1.00 pp) but their position in the *A*. *felis*-clade was unresolved, and DTO 131-E5 was positioned in a clade with moderate statistical support (73% bs, < 0.95 pp) related to the types for *A*. *pseudofelis* (CBS 140763) and *A*. *felis* (CBS 130245).

## Taxonomy

### Morphological and physiological characterization

**Species description of *A***. ***frankstonensis* (CBS 142233 = DTO 341-E7 = IBT 34172; CBS 142234 = DTO 341-F3 = IBT 34204)**

**Fig 3:**
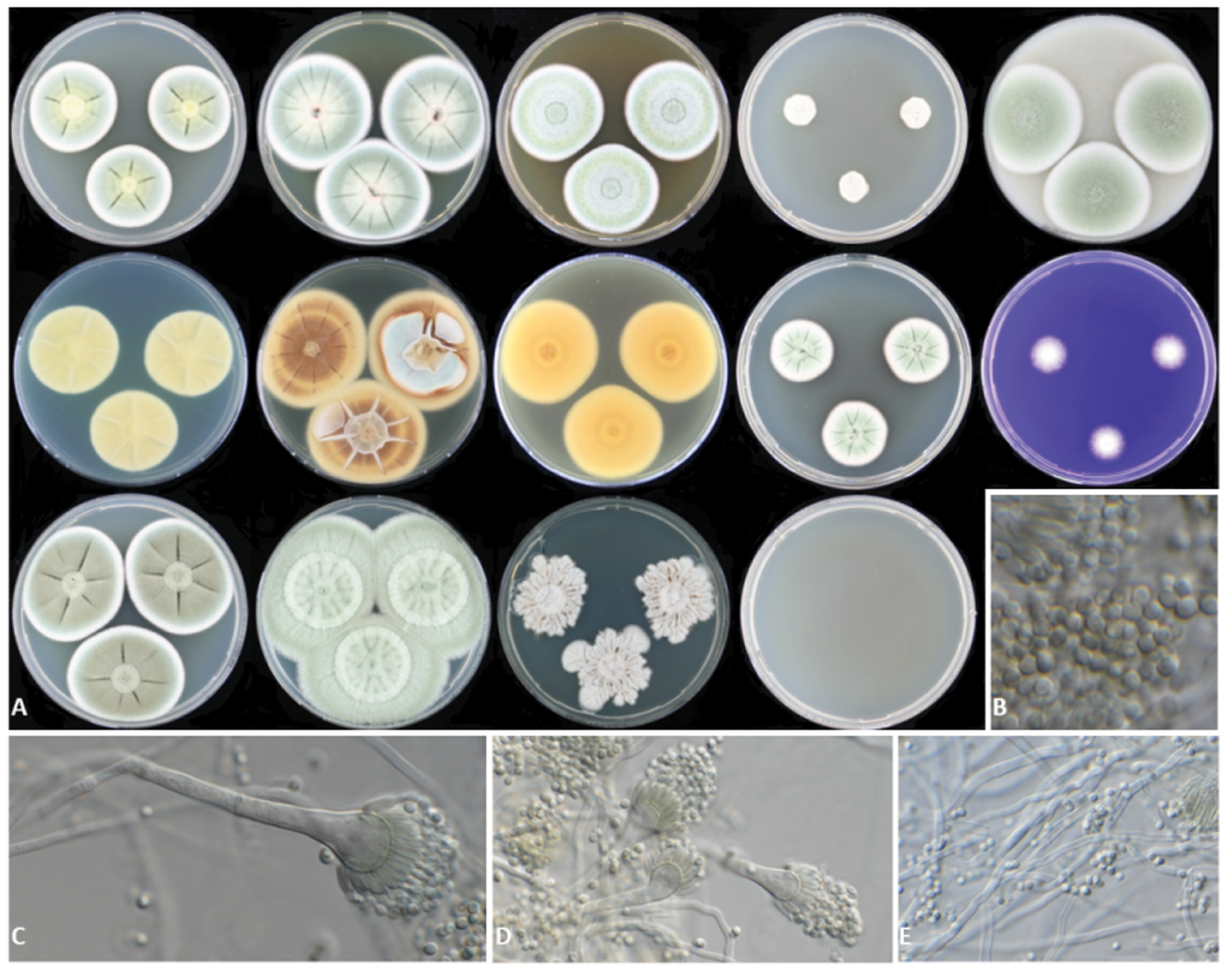
*Aspergillus frankstonensis* CBS 142233; CBS 142234. (A) Colonies grown at 25°C for 7 days, from left to right (top row) CYA, YES, MEA, CYAS, OA; middle row CYA reverse, YES reverse, MEA reverse, DG18, CREA; bottom CYA grown at 30°C, 37°C, 45°C, 50°C. (B) Conidia (C—D) Conidiophores (E) Hyphae.

***Aspergillus frankstonensis*** Talbot et al 2017, **sp. nov.** MycoBank 819986. Fig 3.

#### Etymology

Named after Frankston, Australia, the collection location of the type strain. This town in Victoria, Australia was also the location where *Aspergillus viridinutans s*. *str*. was first isolated [16].

#### Diagnostic characteristics

*Aspergillus frankstonensis* belongs in *Aspergillus* subgenus *Fumigati* section *Fumigati* and is phenotypically similar to other members of the AVSC as it is generally slow to sporulate and thermophilic. The species is phylogenetically most closely related to *A*. *virdinutans s*. *str*. but differs from this species by its ability to sporulate well at 37°C and grow at 45°C.

#### Specimen examined

**Australia** (latitude 38.1414°S, longitude 145.1225°E), from soil, collection date May 7^th^ 2015, J. Talbot & V. Barrs, (holotype **CBS-H-22969**, culture ex-type CBS 142233 = DTO 341-E7; **Australia**, identical collection information as CBS 142233, CBS 142234 = DTO 341-F3).

Description (Fig 3)

Colony diam, 7 days, 25°C (mm): CYA 30–43; MEA 28–34; YES 42–55; DG18 40–45; CYAS 18–22; OAT 40–43; DG18 20–24; CREA 10–27, poor growth, no acid production. Other incubation temperatures: CYA 30°C 42–53; CYA 37°C 45–56; CYA 45°C 25; CYA 50°C no growth; optimum growth temperature 37°C, maximum between 45 and 50°C.

Macromorphology: CYA 25°C, 7 days: colony sulcate, radiating and concentric patterns; sporulation moderate; colony texture velvety to floccose; conidia yellow-green in centre of colony, dull green towards edge; non-sporulating edge 2 mm; mycelium white to yellow; soluble pigment present, yellow; exudate present as yellow droplets; margin regular; reverse yellow.

MEA 25°C, 7 days: colony slightly raised, sulcate, radiating; sporulation poor; colony texture velvety to floccose; mycelium white to pale yellow; conidia pale to bright green; exudate present as clear to yellow droplets; reverse yellow to orange. YES 25°C, 7 days: colony sulcate; sporulation moderate; mycelium white; conidia *en masse* pale-dull green; non-sporulating edge 3 mm; soluble pigments orange; exudate absent; reverse brown and yellow. DG18 25°C, 7 days: colony sulcate; sporulation moderate, conidia dull green, mycelium white with pink tinge; soluble pigment present, pink to yellow; exudate absent, reverse yellow to brown. OA 25°C, 7 days: colony elevated; sporulation moderate; colony texture floccose; mycelium white; conidia pale green; soluble pigment absent; exudate absent; sclerotia absent; reverse yellow.

Micromorphology: Conidial heads columnar, uniseriate. Stipes hyaline, smooth walled, 60–130 × 4–5 μm. Vesicles subglobose, up to 12.5 μm in diameter. Phialides ampulliform, 6–8 × 2–3 μm, covering ∼75% of the head. Conidia globose, smooth, hyaline 2 × 2–3 μm; average width/length = 0.95, n = 40; Hülle cells absent.

#### Occurrence

This species has been found in the soil at a recreational reserve, Upper Sweetwater Creek

Reserve in Frankston, Victoria, Australia, 38.1580° S, 145.1350° E.

Genbank accession numbers: CBS 142233: KY808756 (ITS); KY808594 (*BenA*); KY808724 (*CaM*); KY808948 (*RPB2*); KY808549 (*Act*); KY808901 (*Mcm7*). CBS 142234: KY808761(ITS); KY808599 (*BenA*); KY808729 (*CaM*); KY808953 (*RPB2*); KY808554 (*Act*); KY808906 (*Mcm7*).

### Secondary metabolite production

Both isolates produced viriditoxin, apolar indole-alkaloids and three new compounds given the temporary names of DOLD, RAIMO and CALBO, based on their unique UV spectra. The CALBO compounds had an absorption maximum at 343 nm quite similar to calbistrins. One of the isolates (CBS 142234) was also observed to produce two chrysogine precursors, a unique apolar indol alkaloid and an additional three new compounds given the temporary names of OKAM, USOC and COT based on their UV spectra. The other isolate (CBS 142233) produced aszonapyrone A & B, a chrysogine precursor, apolar indolalkaloids and an additional two compounds given the temporary name of HITO and BRUDA. *Aspergillus frankstonensis* shares viriditoxin production with *A*. *viridinutans*, and aszonapyrones with several *Aspergillus* section *Fumigati* species.

### Antifungal susceptibilities

Antifungal susceptibility results are summarized in Table 2 for two *A*. *frankstonensis* isolates and seven *A*. *felis-*clade isolates (6 environmental, 1 clinical). There was no observed activity of ITZ against one environmental *A*. *felis-*clade isolate.

**Table 2:** Antifungal susceptibility results for two *A*. *frankstonensis* isolates, six *A*. *felis-*clade isolates from environmental soil and one *A*. *felis-*clade clinical isolate (cat). MIC/MEC (μg/mL) values reflect the number of isolates within the specific cut-off value.

Drug	Species	MIC/MEC (μg/mL) distribution among tested isolates												
		0.015	0.03	0.06	0.12	0.25	0.5	1	2	4	8	16	>16	GM
AMB*	*A*. *frankstonensis*				1			1						0.35
	*A*. *felis-*clade							1	5	1				2.00
ITZ*	*A*. *frankstonensis*				1		1							0.24
	*A*. *felis-*clade							4	2				1	1.26
VCZ*	*A*. *frankstonensis*							1		1				2.00
	*A*. *felis-*clade							1	2	4				2.69
POS*	*A*. *frankstonensis*					1	1							0.35
	*A*. *felis-*clade						1	6						0.90
**CSP**†	*A*. *frankstonensis*	1	1											0.02
	*A*. *felis-*clade	5	1	1										0.02

*MIC, minimum inhibitory concentration;

^†^MEC, minimum effective concentration

### Mating type analysis

**Fig 4:**
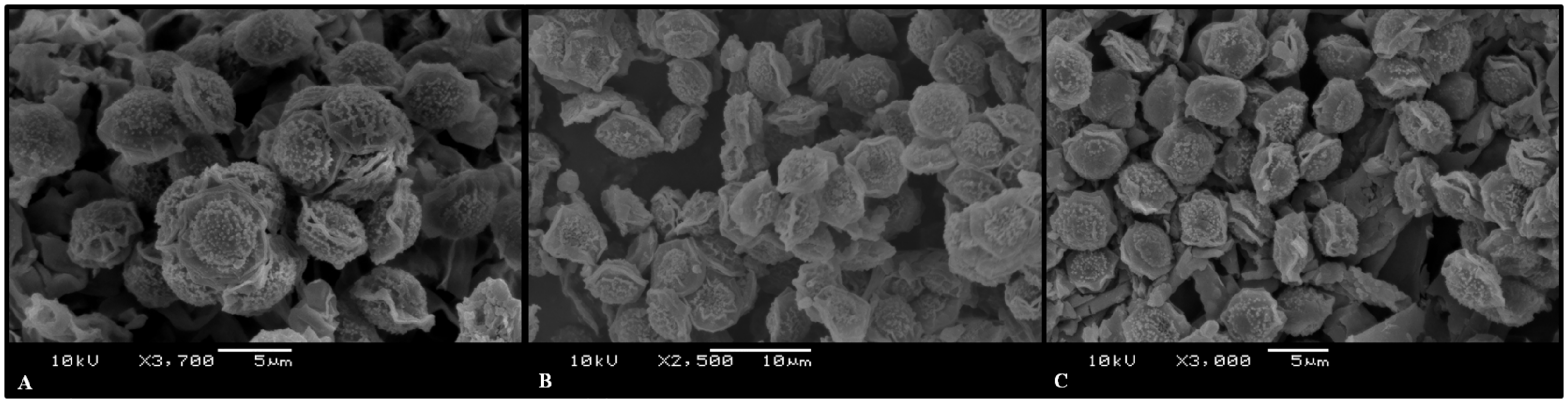
Ascospores from mating experiments with *A*. *felis-*clade isolates. A = *A*. *felis-*clade (DTO 341-E6) X *A*. *felis-*clade (DTO 341-E5); B = *A*. *felis-*clade (DTO 341-E6) X *A*. *wyomingensis* (CCF 4416; C = *A*. *felis-*clade (DTO 341-F2) X *A*. *felis* (DTO 131-E3).

Both isolates of *A*. *frankstonensis* sp. nov. were MAT1-2. All pairings with MAT1-2 isolates of other AVSC species were negative (supplementary Table 2). Both mating types MAT1-1 (n = 5) and MAT1-2 (n = 2) were found amongst *A*. *felis-*clade environmental isolates. Positive intra-species and inter-species matings between opposite mating types (Table 3) produced clusters of white to creamish cleistothecia along the barrage zone that contained lenticular ascospores with two prominent equatorial crests and an echinulate convex surface. Ascospores from three *A*. *felis-*clade intra-species and one inter- species pairing with *A*. *felis* were fertile and from one pairing with *A*. *wyomingensis* were infertile (Fig 4). No growth was seen from parental strains.

**Table 3:** Intra- and interspecific mating results among environmental *A*. *felis-*clade isolates.

	MAT1-1 strain						
	*A*. *felis*-clade	*A*. *felis*-clade	*A*. *felis*-clade	*A*. *felis*-clade	*A*. *felis*-clade	*A*. *felis*	*A*. *felis*
MAT1-2 strain	DTO 341-F1	DTO 341-E6	DTO 341-E4	DTO 341-E8	DTO 341-E9	DTO 131-E3	DTO 131-E5
***A*. *felis-clade*** **DTO 341-F2**	+	+*	-	-	+	+	+
***A*. *felis-*clade** **DTO 341-E5**	+*	+*	-	-	-	-	-
***A*. *felis*** **DTO 131-E4**	-	+*	+	-	-	-	-
***A*. *wyomingensis*** **CCF 4416**	-	+	-	-	-	-	-
***A*. *frankstonensis*** **DTO 341-F3**	-	-	-	-	-	-	-

^-^ no cleistothecia or ascospores;

^+^ cleistothecia production;

*fertile ascospores

### Taxonomic notes

*Aspergillus frankstonensis* has some unique morphological characteristics that can be used to further distinguish it from its closest AVSC relatives (including *A*. *viridinutans s*. *str*., *A*. *arcoverdensis* and *A*. *udagawae*). Grossly, when grown on CYA at 25°C in the dark for 7 days, *A*. *frankstonensis* has a smaller colony diameter than *A*. *arcoverdensis* and *A*. *udagawae* (*A*. *frankstonensis* 30–43 mm; *A*. *arcoverdensis* 56–58 mm; *A*. *udagawae* 82–85 mm), and a larger colony diameter than *A*. *viridinutans* (28–40 mm) [14, 33]. On MEA *A*. *frankstonensis* has pale to bright green conidia on white to pale yellow mycelium, whereas its closely related species range from yellowish white (*A*. *arcoverdensis*) to gray green (*A*. *viridinutans*) to dull green (*A*. *udagawae*) [33]. Microscopically, the conidial head of *A*. *frankstonensis* (60–130 × 4–5 μm) differs in size compared to *A*. *arcoverdensis* (82–110 × 22.5–30 μm), *A*. *viridinutans* (50 × 30 μm) and *A*. *udagawae* (95–145 × 20–50 μm) [33]. It differs from *A*. *fumigatus* by its inability to grow at 50°C.

## Discussion

Our findings suggest that human and animal exposure to pathogenic *Aspergillus* species in Australia is not uncommon since 25% of all ASF isolates were pathogenic species. As expected, the predominant *Aspergillus* species isolated was *A*. *fumigatus s*. *str*. and this species remains the most common cause of aspergillosis in humans and animals. However, the incidence of aspergillosis due to other species in ASF is increasing, reported between 3 to 5% of aspergillosis cases in human patients [34–38].

In soil from residential environments we found the human and animal pathogens *A*. *fumigatus*, *A*. *fischeri*, *A*. *laciniosus*, *A*. *lentulus*, *A*. *fumisynnematus*, *A*. *hiratsukae* and *A*. *thermomutatus* (syn. *N*. *pseudofischeri*). Four of these (*A*. *fumigatus*., *A*. *fischeri*, *A*. *lentulus* and *A*. *thermomutatus*) cause IFI in cats [4, 10]. Isolation of these known feline pathogens from soil in areas cats had access to supports that the source of feline infections is environmental. *A*. *felis* conidia have also been found in air [4], making infection of cats possible via disruption of soil or wind dispersal, such as could occur through the natural feline behaviours of digging, sniffing and grooming. Given that infection often occurs in brachycephalic purebred cats of Persian lineage [4, 9], an immunogenetic defect may predispose cats to disease [10, 39].

Interestingly, we only isolated AVSC species from soil from Frankston, but not from residential environments, cat litter or cat food. *A*. *viridinutans s*. *str*., was discovered in Frankston in 1954 in rabbit dung and sandy soil [16]. This suggests a possible ecological niche for AVSC species at this site associated with faunae, flora and associated soils [40], or local processes such as bush regeneration and back-burning which may remove competing microbiota [41, 42]. AVSC species have been found to be abundant in other specific regions, including a coal mining reclamation site in Wyoming, USA [14].

The most abundant AVSC species isolated from Frankston were in the *A-felis-*clade, closely related to the type of *A*. *parafelis*, CBS 140762, which is a clinical isolate from the oropharyngeal exudate of a human [8]. Here, we also identified another closely related isolate, OHIGB6-A1, which was cultured from a cat with sino-orbital aspergillosis.

Our soil isolation technique was adapted from two previous studies [14, 18]. As *A*. *felis* is a heterothallic species producing heat resistant ascospores, we aimed to recover activated ascospores by heat treating soil. Soil processed without heat did not recover any members of the AVSC. Thus, the ascospores of these species are present in soil and heat activation appears to be an important surveillance technique for heterothallic AVSC species.

Following their polyphasic taxonomical analysis of 11 AVSC isolates, Sugui et al (2014) described three phylogenetically closely related but separate species within the *A*. *felis*-clade; the already described *A*. *felis* [4] and two new species, *A*. *parafelis* and *A*. *pseudofelis*. We investigated the phylogenetic relationships of 56 clinical and environmental AVSC isolates (Fig 1). Our phylogenetic data, based on the single gene and a combined dataset of six genes demonstrated that *A*. *felis* and *A*. *pseudofelis* are the same species. The distinctiveness of *A*. *parafelis* is questionable due to the positioning of a clade of previously described *A*. *felis* isolates DTO 131 E4, DTO 131-E5 and DTO 131-G1 [4]. These isolates have in the combined dataset a basal position to the *A*. *felis*-clade, but the position of these isolates in the single gene phylogenies isn’t congruent. This data indicates that *A*. *parafelis* is also conspecific with *A*. *felis*, when the gene concordance phylogenetic species recognition concept is applied. This is supported by mating experiments and isolates from different lineages are all able to mate [8]. We demonstrated fertile mating between *A*. *felis* (DTO 131-F4) and an *A*. *felis-*clade isolate (DTO 341-E6), which is closely related to the type strain for *A*. *parafelis* (CBS 140762).

Our phylogenetic data also showed that isolate CBS 458.75 is phylogenetically most closely related to *A*. *pseudoviridinutans*. Investigation into exometabolite production will further determine the relationship, however preliminary studies have shown CBS 458.75 is able to produce some of the same exometabolites that *A*. *pseudoviridinutans* strains (DTO 303-A1, NRRL 6106) produce, including antafumicins, clavatols, fumigatin, VERN and pseurotins.

Here we reported the discovery of a new AVSC species, *A*. *frankstonensis*, which is of unknown pathogenicity. However, the ability to sporulate at 37°C indicate pathogenic potential [1]. Although MICs of most antifungals tested were generally low, one *A*. *frankstonensis* isolate had a high MIC of VCZ (4 ug/mL).

We performed AVSC inter-species mating experiments on *A*. *frankstonensis* sp. nov. as other AVSC members have been reported to mate with other species in the complex [8]. *A*. *frankstonensis* inter-species mating were negative, further confirming its status as a distinct species. Intraspecies mating tests could not be performed as we had only one mating type for *A*. *frankstonensis*. Heterothallism (sexual reproduction) allows genetic recombination and has the potential to increase fitness. This may be beneficial for adaptation to environmental conditions, and may also contribute to drug resistance [43]. Interestingly, the majority of the 19 known pathogenic fungi from the genus *Aspergillus* are also heterothallic, with the exception of some doubtful species, *A*. *beijingensis*, *A*. *qizutongi* and *A*. *wanduanglii* [44]. However, many heterothallic species of unknown pathogenicity also exist.

Recent studies have demonstrated that the small molecule extrolite (secondary metabolite), profiles of ASF species can determine the relatedness and identification of a species [45], and may also predict the potential pathogenicity of a new species where only environmental isolates have been discovered [1]. Extrolite production by AVSC environmental isolates in this study shared similarities with other members in the complex. All isolates were shown to produce viriditoxin, which is produced by all other members of the AVSC and one other ASF species [1], therefore its link to pathogenicity is unknown. The only other ASF species reported to produce viriditoxin is *A*. *denticulatus* [46]. There were some secondary metabolite differences between the two *A*. *frankstonensis sp*. *nov*. isolates from Frankston soil. One isolate produced aszonapyrones and chrysogine precursors which may be associated with pathogenicity [47, 48]. Aszonapyrones have antibacterial properties [47]; and chrysogine is an alkaloid [48]. There was a notable difference in the degree of sporulation between the two isolates with one demonstrating poor sporulation. This may account for the differences in extrolite profiles. However, phylogenetically they are the same species with no nucleotide differences between them, based on a number of targeted genes. Novel extrolites were also produced by these isolates, some of which were shared. These unique extrolites may be produced for fungal competitiveness in the primary habitat of this fungal species. Further analysis of the novel compounds and secondary metabolite profiling of other members of the AVSC will be undertaken by the authors for further comparison between environmental and pathogenic strains.

## Conclusions

The risk of exposure to pathogenic species in ASF in Australia appears to be high. The risk of direct environmental exposure to the AVSC in areas where humans and cats co-habitate in Australia, however appears to be low. There was no evidence of an environmental reservoir of these organisms in the homes of any cats diagnosed with aspergillosis. Detection of AVSC organisms from only one location suggests a niche for these species that favours specific environmental conditions. *A*. *frankstonensis* sp. nov is an interesting new species in ASF that is closely related to known human and animal pathogens and possesses some virulent characteristics including growth at 37°C and a high MIC of voriconazole. It also produces unique secondary metabolites that require further investigation.

## Supporting information

S1 Fig Phylogenetic trees of 104 environmental isolates and type strains for *A*. section *Fumigati* based on *BenA* sequencing (phylogeny model Kimura 2, gamma distribution, 1000 bootstrap replicates). (ZIP)

S1 Table *Aspergillus* sect. *Fumigati* species isolated from air and diluted, heat-treated soil from eight Australian properties where cats were domiciled with confirmed feline upper respiratory tract aspergillosis due to AVSC and one National Park site. (#), number of isolates found at property.
(PDF)

S2 Table AVSC species isolates that were paired with *A*. *frankstonensis* sp. nov. DTO 341-F3 (MAT1-2). (PDF)
