# Supplementary material for: Discovery of Aspergillus frankstonensis sp. nov. during environmental sampling for animal and human fungal pathogens
Source: PLoS One. 2017 Aug 9;12(8):e0181660. doi: 10.1371/journal.pone.0181660 (PMC5549889; doi:10.1371/journal.pone.0181660)
Supplement: S1 Table — (#), number of isolates found at property. (PDF) [file pone.0181660.s002.pdf]

**Property 1 (Leichhardt, NSW)**	**Property 2 (Lane Cove, NSW)**	**Property 3 (Anna Bay, NSW)**	**Property 4 (Russel Lea, NSW)**	**Property 5 (Amaroo, ACT)**	**Property 6 (Miranda, NSW)**	**Property 7 (Lara, VIC)**	**Property 8 (Kealba, VIC)**	**Reserve site (Frankston, VIC)**
A. fumigatus (5)<br>*A. fischeri* (5)<br>*A. laciniosus* (2)	*A. fumigatus* (4)<br>*A. lentulus* (1)	*A. fumigatus* (1)<br>*A. fumisynnematus* (4)<br>*A. fennelliae* (1)	*A. fumigatus* (3)<br>*A. fischeri* (1)<br>*A. lentulus* (1)<br>*A. laciniosus* (1)<br>*A. marvanovae* (3)<br>*A. nishimurae* (6)	*A. laciniosus* (2)<br>*A. hiratsukae* (1)	*A. fumigatus* (6)	*A. fumigatus* (5)	*A. thermomutatus* (1)<br>*A. fumigatus* (27)<br>*A. marvanovae* (2)	*A. fumigatus* (13)<br>*A. felis*-clade (7)<br>*A. frankstonensis* (2)
