# Supplementary material for: Discovery of Aspergillus frankstonensis sp. nov. during environmental sampling for animal and human fungal pathogens
Source: PLoS One. 2017 Aug 9;12(8):e0181660. doi: 10.1371/journal.pone.0181660 (PMC5549889; doi:10.1371/journal.pone.0181660)
Supplement: S2 Table — (PDF) [file pone.0181660.s003.pdf]

**Mating type**	**Species**	**ID number**
MAT1-1	*A. felis*-clade	DTO 341-F1
MAT1-1	*A. felis*-clade	DTO 341-E6
MAT1-1	*A. felis*-clade	DTO 341-E4
MAT1-1	*A. felis*-clade	DTO 341-E8
MAT1-1	*A. felis*-clade	DTO 341-E9
MAT1-1	*A. felis*	DTO 131-E3
MAT1-1	*A. felis*	DTO 131-E5
MAT1-1	*A. viridinutans*	IFM 47045
MAT1-1	*A. udagawae*	IFM 46972
MAT1-1	*A. arcoverdensis*	IFM 61334
MAT1-1	*A. felis*-clade	NRRL 62901
